# HIG1 domain family member 1A disrupts proliferation, migration, and invasion of colon adenocarcinoma cells

**DOI:** 10.1080/21655979.2021.1999368

**Published:** 2021-12-29

**Authors:** Zhenyu Xu, Junjie Sun, Yong Mao, Yang Chen, Ting Zhang, Yan Qin, Dong Hua

**Affiliations:** a Department of Oncology, The Second Affiliated Hospital of Soochow University, Suzhou, Jiangsu, China; b Department of Oncology, The Affiliated Hospital of Jiangnan University, Wuxi, Jiangsu, China; c Department of Pharmacy, The Affiliated Hospital of Jiangnan University, Wuxi, Jiangsu, China; d Department of Pathology, The Affiliated Hospital of Jiangnan University, Wuxi, Jiangsu, China; e Department of Oncology, The Affiliated Wuxi People’s Hospital of Nanjing Medical University, Wuxi, Jiangsu, China

**Keywords:** HIG1 domain family member 1A, colorectal cancer, proliferation

## Abstract

HIG1 domain family member 1A (Higd-1a) interacts with dynamin-like 120 kDa protein to maintain the morphological and functional integrity of the mitochondria and thus plays an important role in the progression of malignant tumors. Higd-1a promotes the proliferation of pancreatic cancer cells and the growth of pancreatic cancer; however, no similar observations have been reported for colorectal cancer (CRC). This study, therefore, aimed to verify the role of Higd-1a in CRC. We downloaded data from the Genotype-Tissue Expression (GTEX) and The Cancer Genome Atlas (TCGA) databases and identified an association between Higd-1a levels in colon adenocarcinoma (COAD) tissues and poor survival using Kaplan–Meier curves. Subsequently, we overexpressed Higd-1a in the human COAD cell line HCT-8, knocked down Higd-1a expression in SW480 cells, and evaluated the effects via quantitative PCR (qPCR) and western blotting. MTT assays, colony formation assay, cell cycle analysis, annexin V-FITC/PI, wound-healing analysis, and transwell assay were used to test cell proliferation, formation of cell colonies, cell cycle progression, migration, invasiveness, and apoptosis. Higd-1a has low transcription levels in COAD tissue and suggests a poor prognosis. Higd-1a overexpression in HCT-8 cells weakened cell proliferation, formation of cell colonies, cell cycle progression, migration ability, and invasiveness, and increased apoptosis. Moreover, the decrease of Higd-1a in SW480 cells induced cell proliferation, formation of cell colonies, cell cycle progression, migration, and invasion, and inhibited apoptosis. Higd-1a is underexpressed in COAD cells and its overexpression impaired the proliferation, migration, and invasiveness of COAD cells.

## Introduction

Colorectal cancer (CRC) is one of the most common cancers in the world and a major cause of cancer-related death [1,2]. Globally, the incidence of CRC in 2018 was 6.1%, ranking fourth among cancers, and the mortality rate was 9.2%, ranking second [3]. The main pathological type of CRC is adenocarcinoma [4–6]. The disease is mainly treated by surgery, and chemotherapy and radiotherapy are used to treat specific subgroups of patients [4]. The disease poses a major threat to health, although the survival rate has increased in recent years [4]. There is evidence that research on CRC can provide more adjuvant treatments suitable for patients with CRC, improve the accuracy of prognosis, and thus directly improve the level of care for those patients [4,7].

Mitochondria are involved in generating energy, maintaining calcium homeostasis and redox homeostasis, and apoptosis, and their activities substantially affect the proliferation of cancer cells [8–15]. In addition, mitochondria are highly dynamic organelles that maintain their morphology and activity through continuous fusion and division [8]. HIG1 domain family member 1A (Higd-1a), located in the mitochondrial inner membrane, negatively regulates the apoptosis process, and mediates the assembly of cytochrome oxidase and the formation of respiratory bodies [8,16–19]. Importantly, Higd-1a interacts with a dynamin-like 120 kDa protein to play a role in mitochondrial fusion and division, and inhibits mitochondrial γ-secretase [8,20–23].

It was found that silencing the Higd-1a gene inhibits the proliferation of pancreatic cancer cells and slows the progress of pancreatic cancer [8]. However, there have been few studies on the role of Higd-1a in colon adenocarcinoma (COAD). Therefore, we hypothesized that changing the level of HIDG-1A would affect the proliferation, migration, and invasion abilities of COAD cells. In order to explore the function of Higd-1a in COAD cells, we studied the effect of overexpression or silencing of Hidg-1a on the biological processes of COAD cells, laying the foundation for further research on the role of Hidg-1a in COAD.

## Methods

### Bioinformatics analysis

RNA-seq datasets of 41 adjacent normal tissues and 473 COAD tissues in Genotype-Tissue Expression (GTEX) and the Cancer Genome Atlas (TCGA) were obtained from UCSC Xena (https://xena.ucsc.edu/) [24] and were used to draw a volcano plot in R Limma package (version 3.8). Subsequently, GraphPad Prism 8 was used to analyze the difference in Higd-1a RNA levels between normal tissues and COAD tissues. Kaplan–Meier (KM) survival curve was drawn in GEPIA 2 (http://gepia2.cancer-pku.cn/#survival), and the group cutoff was selected median.

### Cell lines and cell culture

The human CRC cell line HCT-116 (Catalog No. CCL-247), and human COAD cell lines HCT-8 (Catalog No. CCL-244) and SW-480 (Catalog No. CCL-228), obtained from the American Type Culture Collection (ATCC, Manassas, VA, 20110 USA), were cultured in McCoy’s 5A Medium (ATCC, 30-2007) with 10% fetal bovine serum (FBS), RPMI-1640 Medium (ATCC, Catalog No. 30–2001) with 10% horse serum and Leibovitz’s L-15 Medium (ATCC, Catalog No. 30-2008) with 10% FBS, respectively. Human normal colon epithelial cell line NCM-460, purchased from Antihela BioTech (Xiamen, Fujian, China), was cultured in DMEM (Gibco, Detroit, MI, USA) with 10% FBS.

### Overexpressing Higd-1a in HCT-8 cells

The vector pCDH-EF1α-MCS-T2A-puro was purchased from Antihela BioTech (Xiamen, Fujian, China) for the preparation of the plasmid encoding the Higd-1a protein (Gene ID: 25,994) and named Higd-1a OE. DNAMAN 10.0 was used to design the corresponding primer sequences as shown below. Forward primer: 5ʹ-TAGAGCTAGCGAATTCATGGAGCAGAAGCTTGTGGAG-3ʹ; reverse primer: 5ʹ-CAGCGGCCGCGGATCCAGGCTTAGGTTTTGCCCAG-3ʹ. 1.2 × 10^6^ cells/well HCT-8 cells in 6-well plate were transfected with Higd-1a OE plasmid (5 µg/well) using Lipofectamine 2000, according to the manufacturer’s protocol. After 48 h, cells were analyzed.

### Knockdown of Higd-1a in SW480 cells

Two small interfering RNAs (siRNA) of Higd-1a, siHigd-1a-1, and siHigd-1a-2 were obtained from GenePharma (Shanghai, China). 1.2 × 10^6^ cells/well SW480 cells in 6-well plate were transfected with siRNA (400 pmol/well) using Lipofectamine 2000, according to the manufacturer’s protocol. After 48 h, cells were analyzed. Nonspecific siRNA was used as a negative control (siNC). The sequences of siRNAs are listed in Table 1.

**Table 1.** The sequences of siRNAs

Name	Sense sequence (5ʹ-3ʹ)	Antisense Sequence (5ʹ-3ʹ)
siHigd-1a-1	GGCUUUGUUGUAGGAGCAAUG	UUGCUCCUACAACAAAGCCUU
siHigd-1a-2	GUUGCAUAUGGAUUAUAUAAA	UAUAUAAUCCAUAUGCAACAA
siNC	UUCUCCGAACGUGUCACGUTT	ACGUGACAGGUUCGGAGAATT

### Quantitative PCR (qPCR)

RNA was isolated from cells using an RNA isolation kit (Sigma, product code: 83,913-1EA) and reverse-transcribed using HiScript II 1st Strand cDNA Synthesis Kit (VAZYME BIOTECH Co., Ltd., catalog number: R101-01/02, Nanjing, Jiangsu, China). qPCR was carried out using SYBR Green Master Mix (VAZYME BIOTECH Co., Ltd., catalog number: Q111-02, Nanjing, Jiangsu, China), and an iQ5 Real-Time PCR Detection System (Bio-Rad Laboratories, Hercules, CA, USA) to determine relative RNA expression levels. Primers for qPCR were designed using DNAMAN 10.0 and are shown in as following. 18S forward primer: 5ʹ-CGACGACCCATTCGAACGTCT-3ʹ; 18S reverse primer: 5ʹ-CTCTCCGGAATCGAACCCTGA-3ʹ; Higd-1a forward primer: 5ʹ-AAGATCAGGGATCAAAACTCATTCG-3ʹ; Higd-1a reverse primer: 5ʹ-TAGTATTTCCCCTGCTCTTCAGTT-3ʹ. cDNA was pre-denatured at 94°C for 30 min, followed by 40 cycles of denaturation at 94°C for 15 s, annealing at 60°C for 20 s, and extension at 72°C for 20 s. Finally, target gene expression was quantified using the 2^−ΔΔCt^ method [25].

### Western blotting

Protein from cells was extracted using the RIPA buffer (Beyotime Institute of Biotechnology, product code: P0013C), was quantitated using a BCA protein concentration determination kit (Beyotime Institute of Biotechnology, product code: P0012S), and 20 µg of protein was separated by electrophoresis. Proteins were transferred to polyvinylidene fluoride (PVDF) membranes (Millipore, product code: IPVH00010), which were blocked with 5% skim milk and then incubated with the primary antibody for 2 h at 25 ℃, followed by incubation with an appropriate secondary antibody for 1 h at room temperature. The primary antibodies were Higd-1a antibody (21,749-1-AP, 1:1,000, Proteintech Group, Inc., Wuhan, China) and GAPDH antibody (10,494-1-AP, 1:20,000, Proteintech Group, Inc.). The secondary antibody was anti-rabbit IgG which is an HRP-linked antibody (7074, 1:2,000, Cell Signaling Technology, Boston, Massachusetts, USA). A typically enhanced chemiluminescent kit (Thermo Fisher Scientific, Inc.) and ImageJ v1.48 (National Institutes of Health) were used for visualizing the membranes and densitometry, respectively, as previously described [26].

### MTT assay

The cells with high expression of Higd-1a or with knockdown of Higd-1a were seeded into 96-well plates with 1.0 × 10^4^ cells per well, respectively. After 24, 48, or 72 h, 20 μL MTT (5 mg/ml) per well was added to do MTT assay as previously described [26]. After the cells were incubated at 37°C for 4 h, 150 μL of DMSO per well was added, and the cell culture plate was shaken for 10 min to dissolve the crystals. Subsequently, the light absorption value of each well was measured at 490 nm on an enzyme-linked immunosorbent detector. The cell growth curve was plotted with time as the abscissa and absorbance as the ordinate.

### Colony formation assay

Cells as described above were separately seeded in 6-well plates with 2.0 × 10^3^ cells per well. After 2 weeks, the cell colonies were stained with 0.5% crystal violet, photographed under a microscope, and counted, as previously described [27].

### Cell cycle assay

Cells as described above were separately seeded at 1.2 × 10^6^ cells per well. After 24 h, cell cycle assay was performed as previously described [27]. In brief, the cells were harvested, fixed in 70% ethanol, and stored at 4°C overnight. After washing twice with PBS, the fixed cells were permeabilized by adding 0.2% Triton X-100 containing 10 μg/mL RNase at 37°C. Cells were stained with propidium iodide (PI, 20 μg/mL) and analyzed using a flow cytometer.

### EdU assay

Cells with high expression of Higd-1a or with knockdown of Higd-1a were evenly spread across 96-well plates. After 24 h, EdU (RiboBio, Guangzhou, Guangdong, China) was used to detect cell proliferation and DNA synthesis according to the manufacturer’s instructions and as previously described [28]. Images were obtained with a fluorescence microscope (MOTIC, Hong Kong, China).

### Cell apoptosis assay

Cells, as described above, were separately seeded at 1.2 × 10^6^ cells per well. After 24 h, adherent and floating cells were collected, washed with PBS, and resuspended in an incubation buffer containing annexin V-FITC and PI (A211-02, Vazyme Biotech Co., Ltd., Nanjing, China) in the dark for 10 min at 28°C, as previously described [27]. Stained cells were analyzed with a flow cytometer.

### Wound-healing assay

Cells as described above were separately seeded at 1.2 × 10^6^ cells per well. Wound-healing assay was performed as previously described [26]. Briefly, after the cells adhered and reached 100% confluence, a straight-line wound was created by scratching with a 2 μL pipette tip. The floating cells were washed away. A serum-free medium was added to continue culturing for 48 h and the culture was photographed under a microscope at 0 and 48 h.

### Transwell assay

Migration was measured using Matrigel-free transwell plates (Corning, Midland, Michigan, USA) with an 8 μm porous membrane, and invasion was measured using transwell plates coated with Matrigel. Cells as described above were separately seeded into the upper chambers of the transwell at a density of 1.0 × 10^5^ cells per well. The subsequent operation follows the previous description [29].

### Statistical analysis

We used SPSS 22.0 for statistical analysis. We performed Mann–Whitney test and Student’s t-test (unpaired) for nonparametric and parametric data between two groups, respectively, the Wilcoxon matched-pairs signed rank test for nonparametric matched samples, and one-way analysis of variance (ANOVA) followed by Tukey’s post-hoc test for comparison among multiple groups. Log-rank test was used for KM survival analysis. We considered values of p < 0.05 to indicate a significant difference.

## Results

Here, we aimed to investigate the role of Higd-1a in COAD cells. By analyzing data from public databases and performing in vitro experiments, we found that overexpression of Higd-1a in HCT-8 cells inhibited cell proliferation, migration, and invasion, and promoted apoptosis, while silencing Higd-1a in SW480 cells increased cell proliferation, migration, and invasion, and suppressed apoptosis. These findings revealed that Higd-1a has the potential to inhibit COAD, laying the foundation for further research on the molecular mechanism of Higd-1a inhibiting COAD.

### Higd-1a is expressed at a low level in COAD cells

**Figure 1.**
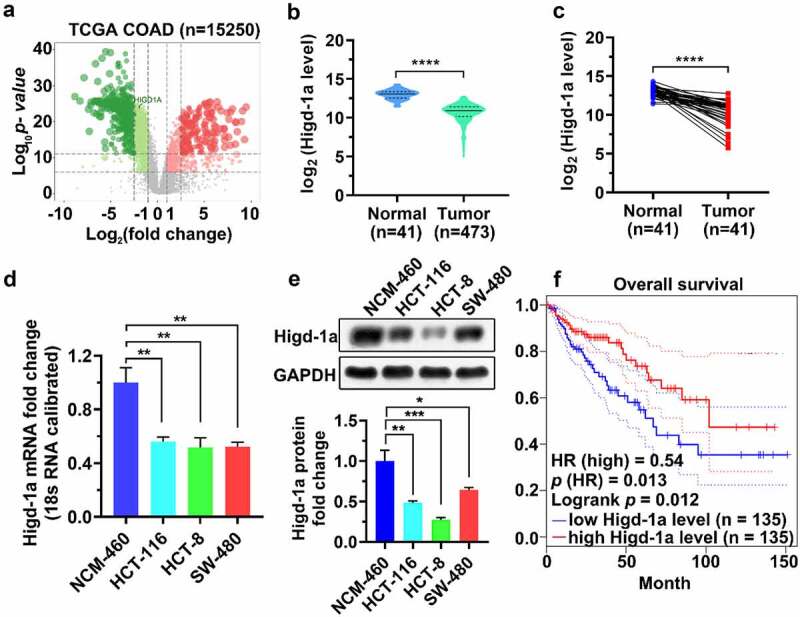
Higd-1a is expressed at a low level in COAD cells.

Analysis of GTEX and TCGA data revealed that the Higd-1a mRNA level in COAD tissues was lower than that in healthy colon tissues (Figure 1a–1c). KM curve analysis showed that the overall survival time of patients in the low Higd-1a level group was shorter than that of patients in the high Higd-1a level group (Figure 1f). In addition, we detected and quantified the mRNA and protein levels of Higd-1a in the COAD cell lines HCT-116, HCT-8, and SW-480, and in the healthy colon epithelial cell line NCM-460 using qPCR and western blotting, respectively, and found that the levels of Higd-1a in the COAD cell lines were lower than that in the NCM-460 line (Figure 1d, 1e). Since the protein level of Higd-1a was lowest in HCT-8 cells (Figure 1e), the HCT-8 cell line was used in the subsequent experiments to establish a Higd-1a overexpressed cell line for further investigation into the effect of Higd-1a on COAD cells.

(a) RNA-seq datasets from TCGA were used to draw a volcano plot of gene expression in patients with COAD with a threshold of |log_2_ fold change| >1 and log_10_ p-value >5 in R Limma package. Red dots represent upregulated genes and green dots represent downregulated genes. (b) The levels of Higd-1a in healthy tissue and COAD tissue from TCGA and GTEX databases were compared using GraphPad Prism 8. (c) The Higd-1a RNA levels in paired adjacent normal tissues and COAD tissues from TCGA were compared using GraphPad Prism 8. (d, e) Higd-1a expression levels in four cell lines, NCM-460, HCT-116, HCT-8, and SW-480, were analyzed by qPCR (d) and western blot analysis (e). (f) The correlation between Higd-1a expression level of patients with COAD and overall survival rate was analyzed via KM curve analysis. The blue and red dotted lines are the 95% CI of the KM curve. TCGA: The Cancer Genome Atlas; COAD: colon Adenocarcinoma; HR: Hazard ratio. Mann–Whitney test (b), Wilcoxon matched-pairs signed rank test (c), one-way ANOVA followed by Tukey’s post-hoc test (d), and Log-rank test (f) were used to analyze the data. *: *p* < 0.05; **: *p* < 0.01; ***: *p* < 0.001; ****: *p* < 0.0001.

### Overexpression of Higd-1a suppresses the proliferation and promotes apoptosis of HCT-8

**Figure 2.**
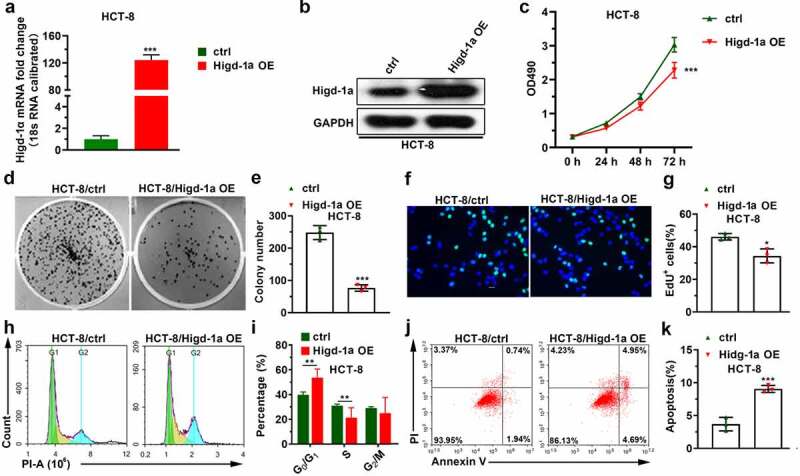
High expression levels of Higd-1a suppress the proliferation of HCT-8.

The increased expression of Higd-1a in overexpressed Higd-1a cells is shown in Figure 2a and 2b. MTT and EdU assays, crystal violet staining, and PI staining were used to detect cell proliferation, colony formation, and cell cycle changes of these cells, respectively. Overexpression of Higd-1a inhibited cell proliferation and reduced the formation of cell colonies (Figure 2c–2g). In addition, the overexpression of Higd-1a arrested the cell cycle in the G0/G1 phase (Figure 2h–2I). These results indicated that high expression of Higd-1a decreased proliferation of HCT-8 cells. Subsequently, we used annexin-V/PI staining together with flow cytometry to detect the apoptosis of HCT-8 cells that overexpressed Higd-1a. Higd-1a overexpression increased apoptosis of HCT-8 cells (Figure 2j, 2k). These results revealed that Higd-1a overexpression promoted apoptosis.

(a, b) Higd-1a expression levels in cells overexpressing Higd-1a (Higd-1a OE) were detected by qPCR (a) and western blotting (b). (c) Effect of high expression of Higd-1a on cell proliferation was analyzed by MTT assay. (d) Effect of overexpressing Higd-1a expression level on cell colony formation was tested by colony formation experiment. (e) Statistical histogram of the number of cell colonies (n = 3). (f) EdU assay was used to test the effect of increasing Higd-1a on cell proliferation. (g) Quantification of EdU-positive cells (n = 3). (h) Effect of upregulating Higd-1a expression level on the cell cycle of HCT-8 cells was detected using PI staining. (i) Statistical histogram of the proportion of cells in different stages of the cell cycle. (j) Annexin V-FITC and PI staining were used to analyze the effect of overexpressing Higd-1a on apoptosis in HCT-8 cells. (k) Statistical histogram of the percentage of cell apoptosis. Student’s t-test (unpaired) (a, c, e, g, i, and k) was used for statistical analysis. Ctrl: HCT-8 cells without higd-1a overexpression; Higd-1a OE: HCT-8 cells overexpressing Higd-1a; PI: propidium iodide; *: *p* < 0.05; **: *p* < 0.01; ***: *p* < 0.001.

### Overexpression of Higd-1a weakens the migration ability and invasiveness of HCT-8

**Figure 3.**
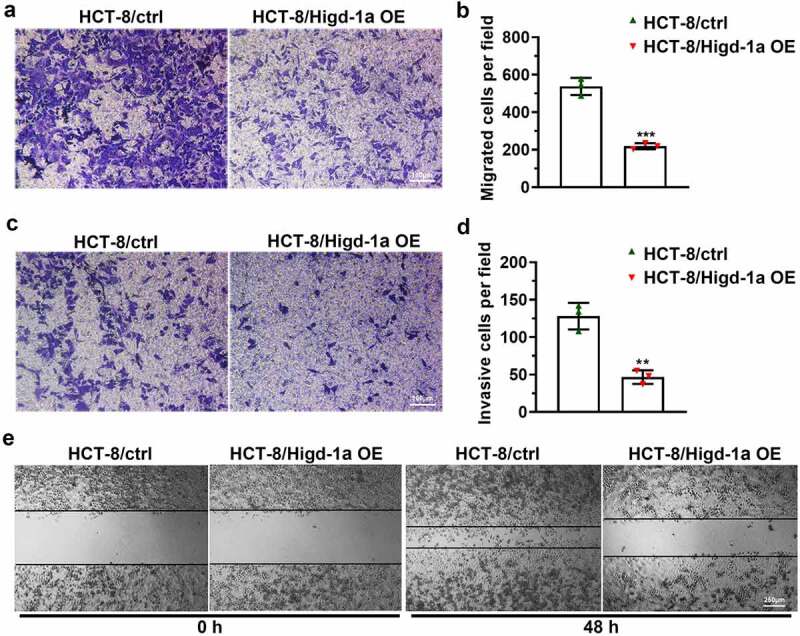
Overexpression of Higd-1a promotes apoptosis and weakens migration ability and invasiveness of HCT-8 cells.

The ability of tumor cells to migrate and invade is the basis of tumor metastasis, and can help the cancer cells spread from the primary tumor and enter the circulatory system [30]. Thus, we tested the changes exerted by the overexpression of Higd-1a on the migration and invasiveness of HCT-8 cells by wound-healing and transwell assays. The results of the transwell assay suggested that high expression of Higd-1a decreased the number of migrated and invasive cells (Figure 3a–3d). Moreover, the overexpression of Higd-1a reduced the wound-healing ability of HCT-8 (Figure 3e). The result indicated that the overexpression of Higd-1a crippled the migration ability and invasiveness of HCT-8 cells.

(a) The results of the Transwell assay without Matrigel showing the function of overexpression of Higd-1a on migration ability in HCT-8 cells. (b) Statistical histogram of the number of migrated HCT-8 cells treated after transwell assay. (c) The results of the Transwell assay with Matrigel showing the invasiveness of HCT-8 cells with a high level of Higd-1a. (d) Statistical histogram of the number of invasive HCT-8 cells following a transwell assay. (e) Wound-healing assay was employed to test the migration ability of HCT-8 cells overexpressing Higd-1a. Student’s t-test (unpaired) (b and d) was used for statistical analysis. Ctrl: HCT-8 cells without higd-1a overexpression; Higd-1a OE: HCT-8 cells overexpressing Higd-1a; **: *p* < 0.01; ***: *p* < 0.001.

### Downregulation of Higd-1a enhances proliferation and inhibits apoptosis of SW480

**Figure 4.**
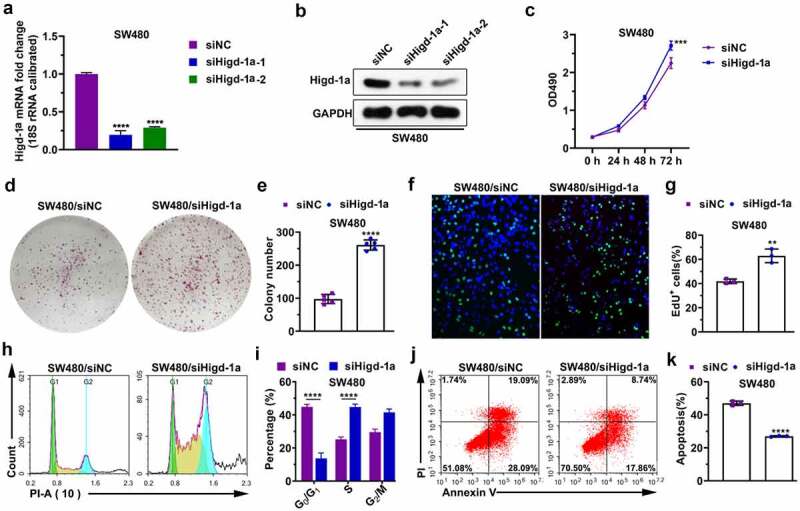
Downregulation of Higd-1a enhances proliferation and inhibits apoptosis of SW480.

To further investigate the role of Higd-1a in colon cancer cells, we downregulated Higd-1a levels in SW480 cells. After SW480 cells were transfected with the siRNAs of Higd-1a, the mRNA and protein levels of Higd-1a were detected by qPCR and western blotting, respectively (Figure 4a, 4b). Decreasing Higd-1a promoted cell proliferation and the formation of cell colonies (Figure 4c–4g). Furthermore, the decrease of Higd-1a induced the transition of cell cycle from G0/G1 phase to S phase (Figure 4h, 4I). These findings suggested that downregulating Higd-1a intensified proliferation of SW480 cells. Next, the apoptosis of SW480 cells lacking Higd-1a was suppressed (Figure 4j, 4k). These data disclosed that decreasing Higd-1a inhibited apoptosis.

(a, b) After Higd-1a was knocked down in SW480 (siHigd-1a-1, siHigd-1a-2), Higd-1a expression levels were detected by qPCR (a) and western blotting (b). ****: *p* < 0.0001 vs. siNC. (c) Effect of low expression of Higd-1a on cell proliferation was analyzed by MTT. ***: *p* < 0.001 vs. siNC. (d) Colony formation assay was used to analyze the effect of downregulating Higd-1a on cell proliferation. (e) Statistical histogram of the number of cell colonies. ****: *p* < 0.0001 vs. siNC. (f) The effect of decreasing Higd-1a on cell proliferation was analyzed using EdU assay. (g) Quantification of EdU-positive cells (n = 3). **: *p* < 0.01 vs. siNC. (h) Effect of silencing Higd-1a on the cell cycle of SW480 was detected using PI staining. (i) Statistical histogram of the proportion of cells in different stages of the cell cycle. ****: *p* < 0.0001 vs. siNC. (j) Representative images showing the effect of downregulating Higd-1a on apoptosis in SW480 cells. (k) Statistical histogram of the percentage of cell apoptosis. ****: *p* < 0.0001 vs. siNC. One-way ANOVA followed by Tukey’s post-hoc test (a) and Student’s t-test (unpaired) (c, e, g, i, and k) was used for statistical analysis. siNC: negative control of siRNA; siHigd-1a: siRNA of Higd-1a; PI: propidium iodide.

### Decreasing Higd-1a induces the migration and invasion of SW480 cells

**Figure 5.**
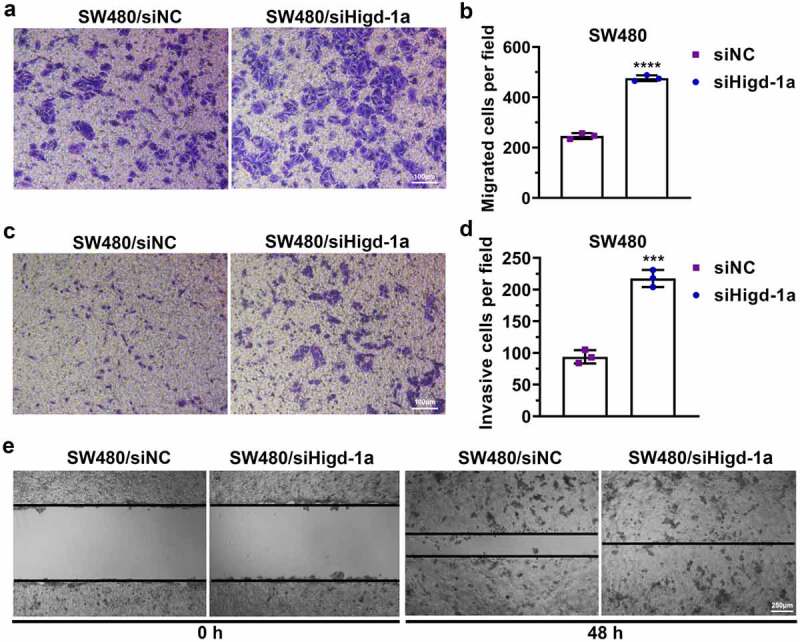
Decreasing Higd-1a induces the migration and invasion of SW480 cells.

The downregulation of HIGD-1A in SW480 cells significantly increased the number of migrated and invaded cells (Figure 5a–5d). Moreover, decreasing Higd-1a improved the wound-healing ability of SW480 (Figure 5e). These results implied that the downregulating Higd-1a promoted the migration ability and invasiveness of SW480 cells.

(a) Representative image of transwell assay without Matrigel showing the function of decreasing Higd-1a on migration ability in SW480 cells. (b) Histogram of the number of migrated SW480 cells. (c) Representative image of transwell assay with Matrigel showing the invasiveness of SW480 cells. (d) Histogram of the number of invasive SW480 cells following a transwell assay. (e) Representative images of wound-healing assay with SW480 cells. Student’s t-test (unpaired) (b and d) was used for statistical analysis. siNC: negative control of siRNA; siHigd-1a: siRNA of Higd-1a; ***: *p* < 0.001; ****: *p* < 0.0001.

## Discussion

CRC is the second leading cause of cancer-related death worldwide [3]. Although the five-year survival rate of patients with early CRC is high, the survival rate of patients with advanced CRC is extremely low, only 8–12% [31,32]. Research into the underlying molecular mechanisms of CRC could help improve screening procedures and patient survival [31]. Currently, cytokeratin-associated protein in cancer, GTPase KRas, cellular tumor antigen p53, and Netrin receptor DCC are considered to promote the progress of CRC [31,33]. In this study, we found that Higd-1a has the potential to inhibit the progression of COAD.

Mitochondria provide energy, are building blocks for new cells, and control redox homeostasis, carcinogenic signals, innate immunity, and apoptosis, which means mitochondria play an important and versatile role in the progression of malignant tumors [9]. Higd-1a interacts with Opal and is necessary for the morphological and functional integrity of mitochondria [21]. Depletion of Higd-1a leads to mitochondrial division, mitochondrial DNA depletion, crest tissue disorder, and growth retardation [21]. Moreover, Higd-1a is induced by hypoxia-inducible factor-1α that plays a major role in the tumor response to hypoxia and contributes to tumor aggressiveness and resistance to radiation therapy and chemotherapy [23,34]. Higd-1a is highly expressed in pancreatic cancer cells whose proliferation is damaged via reducing the expression of Higd-1a [8]. However, in this study, we found that Higd-1a transcriptional levels were lower in COAD tissues than in adjacent normal tissues. Similarly, in COAD cell lines, Higd-1a expression levels were lower than in normal colorectal epithelium. Moreover, the overexpression of Higd-1a reduced the proliferation of COAD cell line HCT-8, while downregulating Higd-1a induced proliferation of COAD cell line SW480. It has been found that Higd-1a, induced and expressed in certain extreme environments lacking HIF, interacts with the mitochondrial electron transport chain to reduce oxygen consumption, thereby achieving the inhibition of cell growth [23]. These results suggest that Higd-1a may play different roles in different tumors.

A study has found that knocking down Higd-1a in pancreatic cancer cells does not increase apoptosis [8]. In addition, it has been shown that induced expression of Higd-1a in extreme HIF-deficient environments reduces the growth of tumors, but promotes the survival of tumor cells *in vivo* [23]. However, in the present study, ectopic expression of Higd-1a in HCT-8 cells decreased proliferation and promoted apoptosis, while silencing Higd-1a in SW-480 cells increased proliferation and inhibited apoptosis. Due to the lack of further research and animal experiments, the mechanism of Higd-1a in COAD remains to be further studied, which is the limitation of this study.

## Conclusion

In conclusion, Higd-1a mRNA is underexpressed in COAD, suggesting a poor prognosis. Overexpression of Higd-1a inhibits the proliferation, migration ability, and invasiveness of HCT-8 cells and promotes apoptosis. Moreover, decreasing Higd-1a improves the proliferation, migration ability, and invasiveness of SW480 cells and suppresses apoptosis. Our findings revealed the function of Higd-1a in COAD cells and lay the foundation for further research on the role of Higd-1a in COAD.
